# The Legacy of COVID-19: Hospital Fear Led to the Delayed Diagnosis of an Ovarian Tumor with Massive Ascites and Extensive Abdominal Necrosis

**DOI:** 10.3390/life15040638

**Published:** 2025-04-11

**Authors:** Janos Szederjesi, Calin Molnar, Claudiu Molnar-Varlam, Dorin Dorobanțu, Mihai Claudiu Pui, Matild Keresztes

**Affiliations:** 1 Department of Anesthesiology and Intensive Care, George Emil Palade University of Medicine, Pharmacy, Science and Technology of Târgu-Mureș, 540139 Târgu Mureș, Romania; yangzi37@gmail.com (J.S.); puimihai@yahoo.com (M.C.P.); 2 Department of General Surgery 1, George Emil Palade University of Medicine, Pharmacy, Science and Technology of Târgu-Mureș, 540139 Târgu Mureș, Romania; molnar.calin@yahoo.com; 3 1st Department of Obstetrics and Gynecology, George Emil Palade University of Medicine, Pharmacy, Science and Technology of Târgu-Mureș, 540139 Târgu Mureș, Romania; molgyn@yahoo.com; 4 Department of Plastic and Reconstructive Microsurgery, George Emil Palade University of Medicine, Pharmacy, Science and Technology of Târgu-Mureș, 540139 Târgu Mureș, Romania; dorindorobantu26@yahoo.com; 5 Department of Anesthesiology and Intensive Care, County Emergency Clinical Hospital of Tîrgu-Mureș, 540136 Târgu Mureș, Romania

**Keywords:** ovarian cancer, massive ascites, abdominal wall necrosis, COVID-19, hospital anxiety

## Abstract

The COVID-19 pandemic has significantly impacted healthcare-seeking behaviors, leading to delayed cancer diagnoses due to hospital-related anxiety. This case highlights the severe consequences of delayed medical consultation in a patient with advanced ovarian cancer. A 47-year-old female presented with severe abdominal distension, massive ascites, and extensive abdominal wall necrosis after avoiding medical care for months due to severe hospital-related anxiety, exacerbated by the loss of her husband during the COVID-19 pandemic. On admission, a CT scan could not be performed due to the patient’s inability to lie supine and extreme abdominal distension. To relieve pressure and improve respiratory function, an abdominal drain was inserted, releasing 72 L of ascitic fluid over five days. Following drainage, imaging confirmed a large ovarian tumor with peritoneal involvement, and a multidisciplinary team (surgeons, gynecologists, plastic surgeons, anesthetists, and intensive care specialists) determined the need for surgical intervention. Histopathology confirmed mucinous adenocarcinoma with pseudomyxoma peritonei (FIGO IIIB). This case underscores the critical impact of delayed oncological diagnosis and the need for enhanced patient education, mental health support, and structured screening programs to prevent similar late-stage presentations.

## 1. Introduction

Ovarian cancer is one of the most common types of cancer in women worldwide and represents the fifth leading cause of cancer-related death among the female population [1,2]. It is often referred to as the “silent killer” because, in most cases, it is detected only in the advanced stages, when survival rates are significantly reduced [3]. Risk factors include a family history of ovarian cancer, the presence of genetic mutations such as BRCA1 and BRCA2, prolonged exposure to estrogen, and infertility [1,2,3,4]. On the other hand, the use of oral contraceptives, multiple pregnancies, and breastfeeding have been shown to have a protective effect [5]. The symptoms of ovarian cancer are often nonspecific and include bloating, abdominal pain, and weight loss [3]. In the advanced stages, severe complications may arise, such as fluid accumulation in the abdomen (ascites), pressure on the organs, and distant metastases [1,4]. In some cases, serious conditions such as tissue necrosis, inferior vena cava compression syndrome, or organ failure may occur, requiring urgent surgical interventions and multidisciplinary coordination [3,6]. Diagnosis of advanced-stage ovarian cancer is based on medical imaging (such as transabdominal or transvaginal ultrasound, computed tomography, and MRI), testing for tumor markers (e.g., CA-125), and tissue biopsy for confirmation [1,2,3,4,5]. The treatment of advanced ovarian cancer generally involves complex surgical procedures to reduce the tumor, followed by adjuvant chemotherapy [3,4,5]. In severe cases, ascites or complications such as abdominal wall necrosis require additional measures, such as draining the accumulated fluid, reconstructive surgical interventions, or other palliative treatments [6,7,8,9,10].

Epithelial ovarian cancer (EOC), the most common ovarian malignancy, is categorized into less-aggressive Type 1 tumors—including low-grade serous carcinoma, endometrioid carcinoma, clear cell carcinoma, and mucinous ovarian carcinoma—and aggressive Type 2 tumors, primarily high-grade serous carcinoma (HGSC).

Mucinous ovarian carcinoma (MOC), the predominant ovarian cancer subtype in women under the age of 40 years, significantly differs from HGSC, as it is not typically associated with risk factors such as BRCA mutations but is notably correlated with tobacco smoking. MOC is characteristically heterogeneous, consisting of benign, borderline, and malignant components; invasive carcinoma is specifically defined by stromal invasion exceeding 5 mm or 10 mm^2^, while invasions below these thresholds are classified as “micro-invasions” within borderline mucinous tumors.

The majority of MOCs (approximately 83%) are diagnosed at an early stage (stage I), conferring a favorable prognosis. In contrast, advanced-stage MOC, though less frequent, has poorer outcomes compared to other epithelial ovarian carcinoma subtypes. The standard treatment approach involves surgical intervention alone for early-stage disease, whereas advanced-stage disease typically requires surgery complemented by adjuvant chemotherapy [11,12].

Romania, although classified as a high-income country, continues to face challenges related to education and health literacy, which significantly affect public health outcomes. These issues were further exacerbated during the COVID-19 pandemic, as limited health literacy and varying levels of education heightened patients’ fears of contracting the virus in medical settings. This fear contributed to substantial delays in seeking medical consultations and attending routine cancer screenings. Additionally, the loss of loved ones during the pandemic profoundly impacted individuals’ trust in healthcare systems and professionals. Personal experiences of bereavement, particularly under restrictive conditions, led to increased skepticism towards medical institutions and practitioners. This erosion of trust further discouraged timely engagement with healthcare services, compounding delays in diagnosis and treatment. Addressing these multifaceted challenges requires targeted efforts to rebuild public confidence through transparent communication, empathetic engagement, and culturally sensitive healthcare practices [13,14,15,16].

## 2. Case Presentation

A 47-year-old female patient presented to the Emergency Department in December 2024 with complaints of dyspnea, diffuse abdominal pain, and progressive abdominal distension, reportedly developing over the preceding six months. Her condition was associated with extensive cutaneous necrosis in the abdominal region, which had emerged two weeks prior and exhibited signs of local infection. Despite the severity of the necrosis, it had neither been documented nor treated. The patient disclosed that she had avoided seeking medical attention for the past three years due to severe hospital-related anxiety, which she later attributed to the traumatic loss of her husband during the COVID-19 pandemic.

The patient lived in an urban area and had completed vocational secondary education (equivalent to high school). She was a homemaker with no formal employment, covered under the national health system as a co-insured beneficiary. This status is granted to spouses or parents without a personal income who are financially supported by an insured family member. Consequently, she did not contribute financially to health insurance but had full access to public healthcare services.

The family history revealed that her daughter had been diagnosed with ovarian cancer and had undergone four surgeries in Romania, followed by another one in Italy, and was currently cancer-free. The patient reported no other relatives known to have cancer. In terms of her medical history, she had hypothyroidism, for which she refused treatment. She denied any prior surgical interventions and reported no known allergies.

The patient was conscious and cooperative during the examination, with spontaneous breathing on ambient air. She exhibited dyspnea upon exertion but remained hemodynamically stable: blood pressure 155/96, heart rate 118, and peripheral oxygen saturation 92% without supplemental oxygen. She compensated well in the sitting position, maintaining adequate respiratory function, but experienced severe respiratory distress (tachypnea, desaturation, panic attack) in supine position.

**Figure 1:**
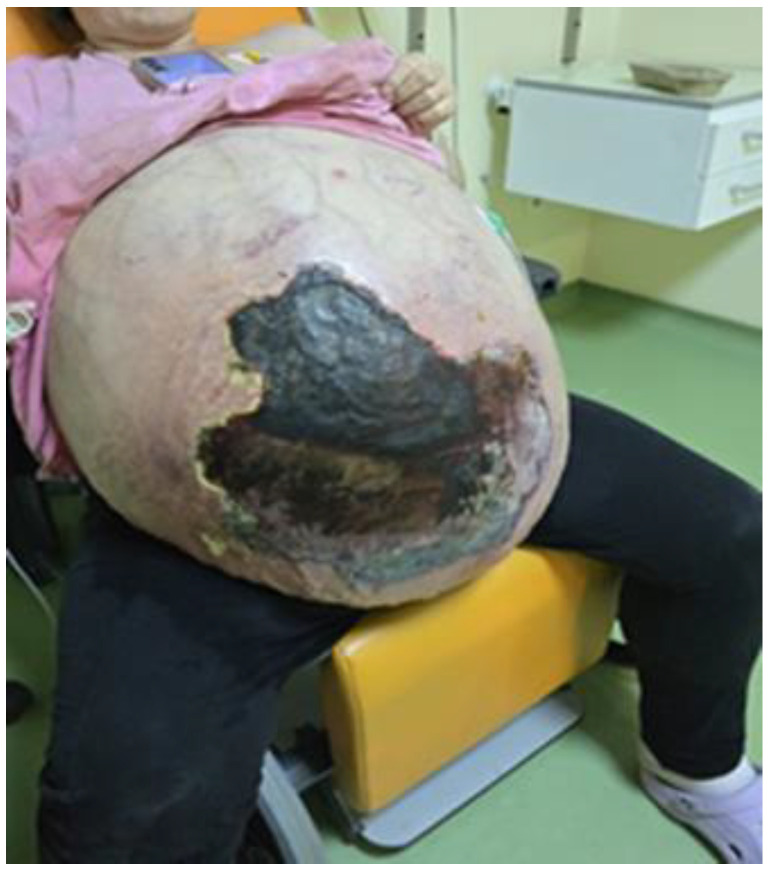
Image of a massively distended abdomen with extensive central necrosis.

Her abdomen was severely distended, extending beyond the xipho-pubic plane and involving the upper abdominal quadrants. On examination, it was diffusely tender, with visible necrosis of the anterior abdominal wall and clinical signs of localized infection. Diuresis was reportedly present, and intestinal transit was faintly perceptible (Figure 1).

An abdominal CT scan was attempted, but it could not be completed due to the patient’s abdominal size and her inability to tolerate the supine position. The latter was primarily attributed to dyspnea caused by her significantly distended abdomen. Abdominal ultrasound was performed but was difficult to interpret because of the massive abdominal distension; however, it revealed significant ascites, adjacent intestinal loops, changes suggestive of a deep intra-abdominal process with a cystic component, and abdominal collateral circulation. An ascitic fluid tap was performed for diagnostic purposes in the Emergency Department, but the gastroenterology team could not perform the paracentesis with the standard kit due to the specific characteristics of the case.

A Multidisciplinary Medical Council was convened, comprising a general surgeon, gastroenterologist, gynecologist, plastic surgeon, and an anesthesia–intensive-care specialist (in Romania, anesthesiology and intensive care are combined into a single specialty). The council decided that the patient should be admitted to the surgical department, and a management plan for the emergency intervention and subsequent care was established.

After being admitted to the surgical department, the patient was transferred to the operating room. Under local anesthesia (lidocaine 1%—total of 300 mg), in a sitting position, the following procedures were performed: paracentesis of the left abdominal flank, placement of triple-circuit abdominal drainage, and extensive debridement of the dry necrosis on the anterior supraumbilical abdominal wall.

Samples of ascitic fluid were collected for both bacteriological and cytological analysis. The bacteriological examination revealed no microbial growth, while cytological evaluation demonstrated moderate leukocytosis.

During the procedure, the patient was monitored and received oxygen at a rate of 5 L/min via a face mask and bolus of 10 mcg sufentanil iv (Sofentyl—Medochemie, Limassol, Cyprus) for supplementary analgesia. After inserting the abdominal drainage system, 21 L of ascitic fluid were gradually drained over 4 h. Over the next four days, 51 L of ascitic fluid was drained slowly in the surgical ward, bringing the total volume since drain placement to 72 L of ascitic fluid, in order to be prepared for surgical intervention and to avoid a high quantity of fluid removal in a short period of time.

No bacteriological analysis was performed, as samples from the necrotic tissue and excised areas were not submitted for microbiological examination.

Blood pressure was monitored throughout the paracentesis, with no episodes of hypotension.

After the placement of a urinary catheter on the day of admission, the patient exhibited oliguria (500 mL/24 h). Following the initiation of paracentesis, urine output increased. During hospitalization on the ward, the patient received intravenous fluids (2000 mL/day of Ringer’s, saline and glucose solutions), ceftriaxone (2 g twice daily), human albumin (20 g/day—local protocol) after the paracentesis procedures, and analgesics on the first day (metamizole 3 × 1 g), along with the oral intake of food and fluids. Ceftriaxone was administered to address the leukocytosis and elevated C-reactive protein (CRP) levels (Table 1).

**Figure 2:**
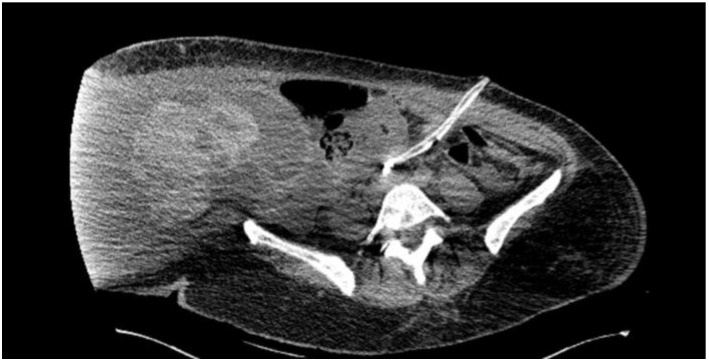
Computer tomography image in axial section plane at the level of the lower abdomen and pelvis revealing a cystic-type tumor.

After the abdominal volume had decreased, the patient was transported to the CT scanner, which revealed the following findings: in the right abdominal flank, a space-occupying lesion was observed. This encapsulated lesion had irregular, well-defined contours, mixed fluid-hemorrhagic densities, and measured approximately 14.6 × 22 × 30 cm (Figure 2). Moderate free peritoneal fluid was present in the abdominopelvic cavity and hepato-splenomegaly was observed. The uterus was anteverted but could not be fully evaluated due to artifacts, appearing without suspicious lesions on the CT. Multiple mesenteric lymph nodes with short axis dimensions of up to 10 mm were noted, associated with mesenteric fat infiltration. Bilateral pleural effusions were present, with a maximum anteroposterior dimension of 14 mm. Additionally, there was an alveolar consolidation in the posterior basal segment of the right lung.

After a multidisciplinary meeting (surgeon, gynecologist, plastic surgeon, anesthetist-intensivist), the necessity of surgical intervention was decided upon to remove the encapsulated process and the patient was taken to the operating room on the sixth day of hospitalization. Laparoscopy was initially chosen as a minimally invasive approach to evaluate surgical operability. If the disease had been determined to be inoperable, the procedure would have been limited to obtaining biopsy samples for histopathological confirmation. Although operability was confirmed, technical challenges encountered during laparoscopy necessitated the conversion to open laparotomy. Notably, laparoscopic trocars were positioned to avoid the necrotic regions of the abdominal wall, and the insufflation pressure was deliberately reduced (12 cm H_2_O) to minimize procedural risk.

**Figure 3:**
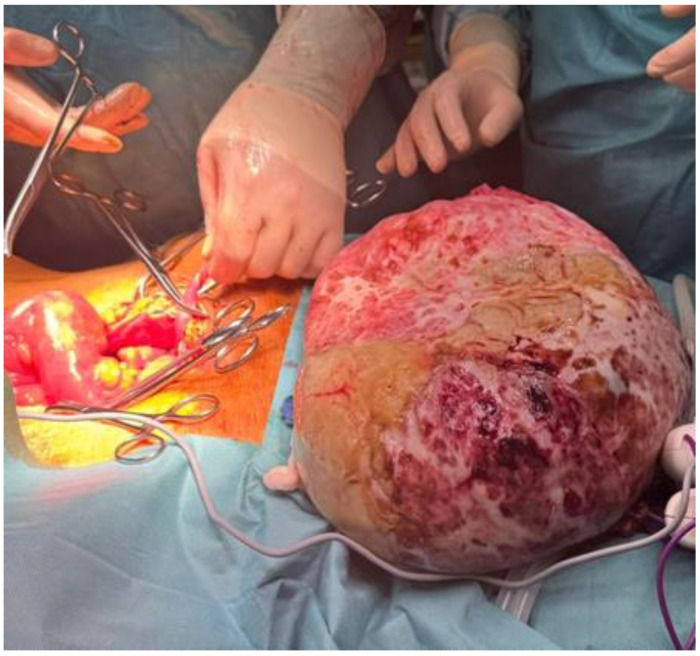
Intraoperative visualization of the resected giant tumor.

Intraoperatively, a cystic-tumor-like formation of approximately 30 × 30 cm was identified, with a whitish appearance, along with multiple necrotic hemorrhagic deposits, false membranes extending from the level of the left ovary, and transmural necrosis due to the compressive effect of the tumor was identified (Figure 3).

During the procedure, a total extracapsular hysterectomy with bilateral salpingo-oophorectomy was performed. A partial resection of the greater omentum and a retrograde appendectomy were also performed, along with extensive debridement. The defect in the skin and soft tissue caused by the debridement was reconstructed using two fascia-cutaneous flaps with superior and inferior pedicles. The presence of massive ascites resulted in considerable abdominal wall distension, which facilitated tension-free reconstruction and enabled abdominoplasty to be performed during the same surgical session.

Biopsy samples were collected intraoperatively and sent for histopathological analysis, including specimens from the left ovary and fallopian tube, uterus, right ovary and fallopian tube, cecal appendix, omentum, and necrotic skin. The surgical procedure lasted five and a half hours. Intraoperatively, the patient lost approximately 2 L of blood (calculated from the hemoglobin drop), for which she received 2 units of red blood cells and 2 units of fresh frozen plasma.

Although the patient initially presented in poor general condition, with massive ascites and marked dyspnea in the supine position, her respiratory status improved significantly following gradual paracentesis. The absence of organ failure and stable hemodynamic parameters supported the decision to proceed with both surgical interventions during a single operative session.

The intraoperative diagnosis included a giant left ovarian tumor, transmural necrosis of the anterior abdominal wall, necrosis of the pouch of Douglas, splenomegaly, and adhesion syndrome.

Postoperatively, the patient was admitted to the intensive care unit, where she remained intubated and mechanically ventilated for 50 min. She was then extubated, maintaining hemodynamic and respiratory stability.

Postoperative intensive care management included iv ceftriaxone 2 × 2 g, iv metronidazole 3 × 500 mg, iv analgesics (paracetamol 3 × 1 g, metamizole 3 × 1 g, nefopam 2 × 40 mg, tramadol 2 × 100 mg), human albumin 20 gr/day, intravenous fluids (NaCl 0.9%/glucose 10%: 2000 mL/day), amino acids (Aminosteril N-Hepa 8% 500 mL/day), and anticoagulation with enoxaparin 2 × 0.4 mL sc. On day 1, oral hydration was initiated. After 4 days in the intensive care unit, the patient recovered without signs of organ dysfunction, leading to the recommendation for transfer to the General Surgery Department.

On the ninth postoperative day, her recovery was progressing favorably, leading to the decision to discharge her. Upon discharge, the patient was conscious, cooperative, afebrile, and hemodynamically stable, with a postoperative wound in the process of healing.

The histopathological results became available approximately two months after the procedure, at which point the patient was referred to a separate oncology hospital for further management.

Histopathological result: microinvasive mucinous adenocarcinoma, most likely originating from the left ovary, associated with pseudomyxoma peritonei involving the pouch of Douglas, omentum, and uterine serosa; stage IIIB (FIGO 2021) [17].

**Figure 4:**
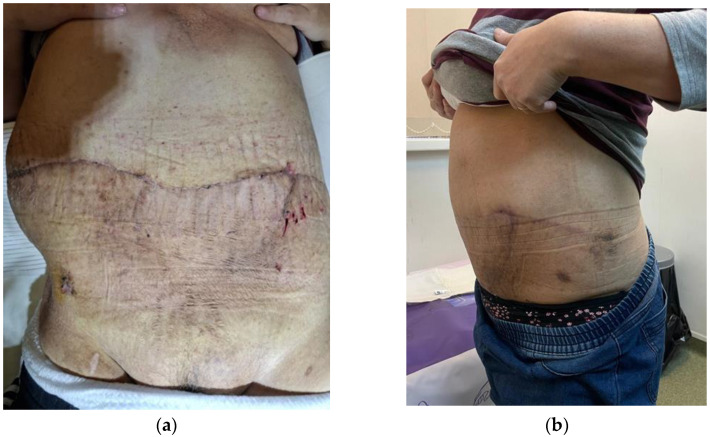
(**a**) The image illustrates the healing process one-month post-surgery, showing significant improvement in tissue recovery; (**b**) healing evolution after two months post-surgery.

The patient demonstrated a favorable wound recovery at 30 and 60 days after surgery (Figure 4).

## 3. Discussion

This case highlights a delayed medical consultation, with the patient presenting with a large, distended abdomen, extensive abdominal wall necrosis, and an intra-abdominal space-occupying lesion. A significant risk factor for ovarian cancer was identified in the patient’s history, as her daughter had previously been diagnosed and treated for ovarian cancer.

In this case, the diagnosis was established based on histopathological findings, as tumor marker testing, including CA-125, was not performed. CA-125 testing is not routinely performed in our hospital; it requires a special request to the laboratory. The test is conducted once a week, and results take 3–4 days to become available.

Given the emergency presentation involving massive ascites and abdominal wall necrosis, the clinical priority was to stabilize the patient and obtain tissue for a definitive diagnosis. Additionally, although the patient had a positive family history—her daughter had previously been treated for ovarian cancer—she had not undergone any prior gynecological evaluation. BRCA1/2 genetic testing was not performed, as this test is not available at our facility and requires referral to an external center with the costs to be covered by the patient, and results typically take 1–2 weeks. In the context of an acute surgical emergency, such testing was not feasible.

In the case presented, the histopathological findings are consistent with the clinical presentation, intraoperative findings, and the patient’s family history.

The rarity of this case lies in the late presentation with extensive abdominal wall necrosis, a condition seldom encountered at such an advanced stage. The delay in seeking medical attention was primarily attributed to the patient’s severe hospital-related anxiety, which was triggered by the loss of her husband in a hospital during the COVID-19 pandemic. In addition, the absence of an effective primary healthcare response and socioeconomic factors—including limited health education and a lack of formal employment—may have further contributed to the delayed consultation and diagnosis.

Romania’s primary healthcare system serves as the initial point of contact for individuals seeking medical assistance, primarily delivered through family physicians operating in private practices contracted by district health insurance funds [18].

In Romania, ovarian cancer screening poses significant challenges due to the absence of standardized protocols and a national screening program. Lack of national screening program for ovarian cancer, leads to inconsistent practices among healthcare providers. Physicians often develop individualized investigation lists, resulting in varied approaches to detection [19].

Fear and anxiety may lead individuals to delay seeking medical care, resulting in advanced disease stages at diagnosis. Studies have shown that patients’ fear of a cancer diagnosis and denial can lead to delays in seeking care, while anxiety about potential side effects and outcomes may cause the postponement of treatment initiation [20].

The COVID-19 pandemic has had a profound and lasting impact on mental health, leading to increased hospital-related anxiety that continues to deter individuals from seeking necessary medical care. Studies have shown that during the pandemic, a significant number of patients delayed or avoided medical consultations, even for serious conditions, due to fear of contracting COVID-19 in hospitals. This avoidance behavior has persisted, leading to delayed diagnoses and treatment [21].

## 4. Conclusions

This case highlights the severe consequences of delayed medical care, exacerbated by hospital-related anxiety.

The late presentation with extensive abdominal wall necrosis and massive ascites underscores the urgent need for early detection strategies to prevent such advanced disease stages. To address these challenges, a structured and comprehensive approach to ovarian cancer screening is essential.

In such advanced cases of ovarian cancer, an early multidisciplinary approach is crucial (upon presentation to the Emergency Department), optimizing the timing of surgical intervention to increase the success rate and prevent potential complications. Admitting these patients to intensive care can help prevent potential complications and enhance their recovery.

## Figures and Tables

**Table 1:** Dynamic laboratory results.

Test	14 December 2024	17 December 2024	18 December 2024	19 December 2024	20 December 2024	Normal Range (Local)	Units
Timeline	Admission	CT Scan		Day of Surgery	Postoperative		
White blood cells	16.15	10.74	8.42	12.40	17.50	3.6–10	10^3^/µL
Red blood cells	5.16	4.34	4.47	3.46	4.19	3.5–5.4	10^6^/µL
Hemoglobin	10.80	9.3	9.3	7.97	9.3	12–16	g/dL
Hematocrit	34.90	28.8	29.6	24.80	29.2	36–48	%
Platelets	484	268	266	275	369	150–450	10^3^/µL
Neutrophils	13.68	8.82	6.69	11.00	14.33	1.4–6.5	10^3^/µL
C-reactive protein	252.80	-	-	76.60	168.00	0–5	mg/L
Direct bilirubin	0.60	0.35	0.26	0.64	0.28	0–0.5	mg/dL
Total bilirubin	1.06	0.42	0.33	-	0.31	0.2–1.2	mg/dL
Creatine phosphokinase total	56.00	118.00	58.50	139.00	743.00	29–168	U/L
Potassium	4.17	3.84	4.23	4.03	4.49	3.5–5.1	mmol/L
Sodium	137.00	131.60	135.00	138.00	137.10	136–145	mmol/L
Urea	21.40	15.70	17.20	19.26	26.20	14.98–40.01	mg/dL
ALT	36.00	22.80	24.50	17.00	33.40	0–55	U/L
AST	48.00	33.80	51.40	31.00	79.20	5–34	U/L
GGT	137.00	87.00	-	75.60	62.40	8–33	U/mL
Creatinine	0.63	0.47	0.41	0.48	0.69	0.57–1.11	mg/dL
eGFR (CKD-EPI-2009)	106.25	117.00	122.38	116.19	103.12	>90	mL/min/1.73 m^2^
Alkaline phosphatase	318.00	189.00	171.00	112.00	106.00	40–150	U/L
Lactate dehydrogenase	235.00	165.00	187.00	179.00	253.00	125–220	UI/L
Albumin	-	-	-	-	2.14	3.97–4.94	g/dL
Total proteins	-	4.19	4.41	-	3.92	6.4–8.3	g/dL

eGFR (CKD-EPI-2009)—Estimated Glomerular Filtration Rate, AST—Aspartate Aminotransferase, GGT—Gamma-Glutamyl Transferase, ALT—Alanine Aminotransferase, 10^3^/µL—thousand cells per microliter, 10^6^/µL—million cells per microliter, g/dL—grams per deciliter, %—percentage, U/L or UI/L—units per liter (or international units per liter), mg/dL—milligrams per deciliter, mg/L—milligrams per liter, mmol/L—millimoles per liter, mL/min/1.73 m^2^—milliliters per minute per 1.73 square meters (body surface area), g/dL—grams per deciliter.

## Data Availability

The data used for this study can be found in the database of the Târgu Mures, County Emergency Clinical Hospital, Romania.

## Abbreviations

The following abbreviations are used in this manuscript:

BRCA	Breast Cancer Gene
CA-125	Cancer antigen
CRP	C-reactive protein
FIGO	International Federation of Gynecology and Obstetrics

## References

[1] Lheureux S., Gourley C., Vergote I., AM O. (2019). Epithelial ovarian cancer. Lancet.

[2] Torre L.A., Trabert B., DeSantis C.E., Miller K.D., Samimi G., Runowicz C.D., Gaudet M.M., Jemal A., Siegel R.L. (2018). Ovarian Cancer Statistics, 2018. CA A Cancer J. Clin..

[3] Ledermann J.A., Raja F.A., Fotopoulou C., Gonzalez-Martin A., Colombo N., Sessa C. (2013). Newly diagnosed and relapsed epithelial ovarian carcinoma: ESMO Clinical Practice Guidelines for diagnosis, treatment, and follow-up. Ann. Oncol..

[4] Prat J., FIGO Committee on Gynecologic Oncology (2014). Staging classification for cancer of the ovary, fallopian tube, and peritoneum. Int. J. Gynecol. Obstet..

[5] Walker M., Sobel M. (2018). Diagnosing ovarian cancer. CMAJ.

[6] Škof E., Merlo S., Pilko G., Kobal B. (2016). The role of neoadjuvant chemotherapy in patients with advanced (stage III C) epithelial ovarian cancer. Radiol. Oncol..

[7] Colombo N., Sessa C., Bois A.D., Ledermann J., McCluggage W.G., McNeish I., Morice P., Pignata S., Ray-Coquard I., Vergote I. (2019). ESMO-ESGO Consensus Conference Recommendations on Ovarian Cancer. Int. J. Gynecol. Cancer.

[8] Banerjee S., Kaye S.B. (2013). New strategies in the treatment of ovarian cancer: Current clinical perspectives and future potential. Clin. Cancer Res..

[9] Cortez A.J., Tudrej P., Kujawa K.A. (2018). Lisowska KM. Advances in ovarian cancer therapy. Cancer Chemother. Pharmacol..

[10] Hennessy B.T., Coleman R.L., Markman M. (2009). Ovarian cancer. Lancet.

[11] Babaier A., Ghatage P. (2020). Mucinous Cancer of the Ovary: Overview and Current Status. Diagnostics.

[12] Poudel D., Acharya K., Poudel N., Adhikari A., Khaniya B., Maskey S. (2022). Bilateral ovarian mucinous carcinoma (stage III) with omental involvement and incidental hydronephrosis: A rare case report. Int. J. Surg. Case Rep..

[13] Popescu A., Craina M., Pantea S., Pirvu C., Chiriac V.D., Marincu I., Bratosin F., Bogdan I., Hosin S., Cosmin C. (2022). COVID-19 Pandemic Effects on Cervical Cancer Diagnosis and Management: A Population-Based Study in Romania. Diagnostics.

[14] Mihai A.M., Ianculescu L., Cretoiu D., Suciu N. (2024). Breast Cancer Screening in Romania: Challenges and Opportunities for Early Detection. Acta Endocrinol. Buchar..

[15] Beller J., Schäfers J., Haier J., Geyer S., Epping J. (2023). Trust in Healthcare during COVID-19 in Europe: Vulnerable groups trust the least. J. Public Health.

[16] Perlis R.H., Ognyanova K., Uslu A., Trujillo K.L., Santillana M., Druckman J.N., Baum M.A., Lazer D. (2024). Trust in Physicians and Hospitals During the COVID-19 Pandemic in a 50-State Survey of US Adults. JAMA Netw. Open..

[17] Berek J.S., Renz M., Kehoe S., Kumar L., Friedlander M. (2021). Cancer of the ovary, fallopian tube, and peritoneum: 2021 update. Int. J. Gynecol. Obstet..

[18] Pavlič D.R., Miftode R., Balan A., Pall Z.F. (2015). Building Primary Care in a Changing Europe: Case Studies.

[19] (2016). FEMALE ONCOLOGIC DISEASES IN ROMANIA, Study Conducted by ISRA Center, for The Coalition for Women’s Health and Roche Romania. https://www.think-pink.be/Portals/0/dtxArt/blok-document/bestand/f05ff8a7-ab9c-47d5-8937-33897a7b9c38.pdf.

[20] Dlamaini X., Hlophe L., Maseko T., Nonhlanhla M., Mandzisi M., Zanele N., Nomxolisi M., Debrah V., Samson H. (2022). Delays to Cancer Care, Exploring the Factors Associated with Barriers to Accessing Comprehensive Cancer Care in Eswatini: A Qualitative Study. Asian Pac. J. Cancer Care.

[21] Yasemin K., Sedat B., Ahmet K., Ömer K., Karataş A., Dereli S. (2021). Effect of COVID-19 pandemic on anxiety depression and intention to go to hospital in chronic patients. Int. J. Clin. Pract..

